# Knowledge, Attitudes, and Practices towards COVID-19 among Pregnant Women in Northern Bangladesh: A Community-Based Cross-Sectional Study

**DOI:** 10.3390/bs13010002

**Published:** 2022-12-20

**Authors:** Homyra Tasnim, Md. Bony Amin, Nitai Roy, Md. Aktarujjaman, Bryan T. Rogers, Raphyel Rosby, Ekhtear Hossain

**Affiliations:** 1 Department of Biological Sciences, Louisiana State University, Baton Rouge, LA 70803, USA; 2 Faculty of Nutrition and Food Science, Patuakhali Science and Technology University, Patuakhali 8602, Bangladesh; 3 Department of Biochemistry and Food Analysis, Patuakhali Science and Technology University, Patuakhali 8602, Bangladesh; 4 Department of Biological Sciences and Chemistry, Southern University and A&M College, Baton Rouge, LA 70813, USA

**Keywords:** COVID-19, knowledge, practice, pregnant women, Bangladesh

## Abstract

Background: COVID-19, caused by SARS-CoV-2, remains a global public health concern despite the availability of effective antiviral treatment against multiple strains. Studies have shown that pregnant women are more susceptible to COVID-19 due to altered physiology and immunological features. Therefore, this study was designed to investigate pregnant women’s knowledge, attitudes, and practice (KAP) to prevent COVID-19 and determine the factors associated with KAP. Methods: A community-based cross-sectional study was conducted among 425 pregnant women in Northern Bangladesh. The samples were obtained using a simple random sampling technique from 5 April to 15 June 2020. The data were collected by face-to-face survey with a structured and pre-tested questionnaire and analyzed using SPSS version 25. Bivariable and multivariable logistic regression analyses were performed, and *p*-values < 0.05 at 95% CI were considered statistically significant. Results: Overall, the score of KAP among the respondents was 47.76%, 49.41%, and 56.24%, respectively. Participants’ area of residence, educational status of the husband, and antenatal care (ANC) visit were significantly associated with the level of knowledge, whereas age, educational status of the husband, number of living children, and knowledge were significant predictors of attitude. The knowledge of COVID-19 was the only predictor associated with the practice. Conclusion: Our study shows that almost half of the participants had poor knowledge, a negative attitude, and poor practices regarding COVID-19. Additional health education programs by healthcare professionals and different media, coordinated and combined efforts of government and individuals’ participation will be required to fight the spread of the infection.

## 1. Introduction

The novel coronavirus disease (COVID-19) is an infectious disease caused by a new type of enveloped RNA coronavirus, severe acute respiratory syndrome coronavirus 2 (SARS-CoV-2) [1]. Since its first report in December 2019 in Wuhan, China, COVID-19 has rapidly evolved with worldwide exponential spread. As a result, the World Health Organization (WHO) declared COVID-19 a global pandemic on 11 March 2020 [2].

Since SARS-CoV-2 strains are constantly evolving, herd immunity has not yet developed, putting all populations at risk of infection. However, as pregnant women are more susceptible to severe infection by respiratory pathogens, they may be more susceptible to COVID-19 infection than the general population [3]. Additionally, the physiological changes and characteristic immune responses of pregnancy leave these women at higher risk from the cytokine storm brought on by COVID-19 infection with the outcome being severe or fatal [4]. For example, a report from Brazil shows that from 26 February 2020 to 18 June 2020, there were 124 maternal deaths due to the pandemic [5]. Furthermore, several studies also reported that COVID-19-positive pregnant women had pre-existing co-morbidity such as diabetic mellitus, bacterial and viral co-infections, and obstetric complications, including premature rupture of membrane, placenta previa, preeclampsia, and postpartum hemorrhage [6]. As additional evidence of their higher risk, the requirement for mechanical ventilation in COVID-19-positive pregnant mothers is higher than that for non-pregnant ones [7].

In addition to the increased risk of the mothers, there is a strong association between COVID-19 and fetal and neonatal complications such as fetal distress, fetal tachycardia, low birth weight, neonatal asphyxia, and stillbirth [8]. Hence, with the ongoing pandemic of COVID-19, both pregnant women and newborn babies should be considered at-risk populations in strategies centering on preventing COVID-19 infection.

A recent study conducted among pregnant women in Ghana found that 85% of the participants had good knowledge of COVID-19 but had poor practice, as 63.4% were not using preventive measures [9]. According to a study on pregnant women in India, the majority of the pregnant women had satisfactory knowledge, positive attitudes, and good practices regarding COVID-19 [10]. Two additional studies from Ethiopia concluded that about half of the pregnant women in their study had poor knowledge and inappropriate practice [11,12]. In these previous studies, the major determinants of knowledge about COVID-19 were women’s age, residence, educational status, occupation, being civil servant, wanted pregnancy, and ANC follow-up, whereas age, educational status, residence, number of children, and knowledge were the major determinants of preventive practices [9,10,11,12]. In addition, age, the participant’s husband’s education, wanted pregnancy, and knowledge were the predictors of attitude [12].

Bangladesh is one of the Asian countries severely affected by the COVID-19 pandemic. In part, this is due to Bangladesh’s lack of experience dealing with epidemics such as SARS or MERS [13] leaving the healthcare system unprepared for the pandemic. The geographic location of Bangladesh, where India surrounds the country from three sides, is also a factor. For example, when the catastrophic surge of delta variants occurred in India during the first part of 2021, it also reached across the border into Bangladesh [14]. Since the COVID-19 outbreak, the government of Bangladesh has undertaken unparalleled measures to control the spread of the virus, including applying public health protocols, such as frequent hand washing, physical distancing, and lockdown measures [14]. Despite all efforts, Bangladesh reported 1,575,185 coronavirus cases and 27,970 deaths through 26 November 2021 [15]. During this time, reports have been published on KAP in Bangladesh focusing on the general population, but data on pregnant women’s KAP remain scarce [14,16,17].

Unfortunately, some rules implemented by the government created public distress and massive fear [18], especially among the unaffected population [19], provoking them to disobey some rules. At the same time, public compliance is crucial for such measures to be effective, and compliance largely depends on their KAP towards COVID-19 [20,21]. Therefore, it is vital to investigate KAP towards COVID-19, and its associated factors, among at-risk populations. We chose pregnant women in Northern Bangladesh for our study. Data obtained from this study will facilitate healthcare professionals’ ability to provide appropriate COVID-19 counseling to women facing the uncertainties of pregnancy during the antenatal, intrapartum, and postpartum periods.

## 2. Methods

### 2.1. Study Design and Setting

This community-based cross-sectional study investigated KAP regarding COVID-19 among pregnant women in Northern Bangladesh. A face-to-face survey was conducted to collect data on the study population from 5 April to 15 June 2020. Five sub-districts were selected randomly from each of the two northern districts, Lalmonirhat and Kurigram: Lalmonirhat Sadar, Kaliganj, Aditmari, Patgram, and Hatibandha from Lalmonirhat district and Kurigram Sadar, Phulbari, Nageshwari, Ulipur, and Rajarhat from Kurigram district. These five sub-districts were chosen for their accessibility under the strict lockdown measures in effect during the survey. Both of these districts are under the Rangpur division, a northern border region of Bangladesh. For data collection, we visited community clinics (primary-level health facilities that the government has established with the participation of local communities), union health and family welfare centers (UH and FHC), and non-governmental organization (NGO) hospitals/clinics in the study areas, while complying with the strict precautionary measures required during the pandemic.

### 2.2. Survey Questionnaire and Tools

Data were collected using face-to-face interview using a structured and pre-tested questionnaire. The questionnaire was first prepared in English, then translated to the local language, Bengali, for understandability, and back to English for consistency. The questionnaire was adapted from WHO guidelines and the relevant literature and modified according to the local context [22]. The questionnaires have six items (socio-demographic characteristics, reproductive health-related characteristics, knowledge-related characteristics, attitude-related characteristics, practice-related characteristics, and source of information regarding COVID-19).

For the knowledge section, each question had a possible response of “Yes” (agree) or “No” (disagree) (e.g., “COVID-19 is a disease caused by a virus.”). The correct answer (Yes) was coded as 1, while the incorrect answer (No) was coded as 0. The total score ranged from 0 to 15, with an overall greater score indicating more accurate knowledge. A score of 0–7 was assigned for a low level of knowledge, and a score of 8–15 was assigned for a high level of knowledge. For attitudes, the questions are answered on a three-item Likert scale consisting of agree, neutral, and disagree options (e.g., “COVID-19 is a deadly disease.”) We assigned 2 points for the first option, 1 point for the second option, and 0 for every third. The total attitude score ranged from 0 to 16, with an overall greater score indicating a more positive attitude. A score of 0–7 was assigned for a negative attitude, and a score of 8–15 was assigned for a positive attitude. In the practice section, questions were answered with “Yes” or “No” (for example, “Are you obeying government restrictions on COVID-19?”). The total score for practice items ranges from 0 to 7, with a higher overall score indicating more accurate practice. A low level of practice was assigned a score of 0–3, and a high level of practice was assigned a score of 4–7.

Pre-testing of the questionnaire was conducted on 10% of the total participants (43 pregnant women) in Lalmonirhat town near the study setting. During the pre-test, the questionnaire was assessed for its clarity, accuracy, comprehensiveness, readability, and optimal time for completing the interview. Modifications and corrections of wording, logical sequence, and skip pattern of the survey were immediately performed based on the pre-test results. Four diploma health professionals (paramedics), familiar with pregnancy, collected the data. Data collectors were trained for one day on the aim of the study, method of data collection, contents of the questionnaire, preserving confidentiality, and receiving informed consent prior to data collection. The completeness and consistency of the collected data were cross-cheeked, cleaned, and compiled by supervisors and principal investigators.

### 2.3. Sample Size Determination and Sampling Procedure

The sample size of 425 participants was calculated using the single proportion formula with the following assumptions: the proportion of KAP of preventive measures against COVID-19 is 50% since there is no related study in Bangladesh, a confidence interval of (CI) 95%, a margin of error (d) 5%, and a non-response rate of 10%. The participants were initially chosen at random from a list of names available at the health facility in our study regions. Only pregnant women who had given informed consent to participate in the study were considered. Those that had antenatal care (ANC) follow-up for current pregnancies were included in the study population while excluding pregnant mothers who had mental problems, hearing difficulties, or were critically ill.

### 2.4. Operational Definitions

Level of knowledge, attitude, and practice were determined using a questionnaire of fifteen knowledge assessments, seven attitude assessments, and eight practice assessments. Results for knowledge and practice were categorized as good or poor based on the mean score. Pregnant women who scored greater than or equal to the mean score of attitude questions toward COVID-19 were considered as having a positive attitude. In contrast, those who scored less than the mean score were considered to have a negative attitude. The calculated mean values for knowledge, attitude, and practice were 7.26, 7.27, and 3.82, respectively.

- Good knowledge: Participants who scored greater than or equal to the mean score.
- Poor knowledge: Participants who scored less than the mean score.
- Positive attitude: Participants who scored greater than or equal to the mean score.
- Negative attitude: Participants who scored less than the mean score.
- Good practice: Participants who scored greater than or equal to the mean score.
- Poor practice: Participants who scored less than the mean score.

### 2.5. Statistical Analysis

Statistical analysis software, IBM SPSS Statistics 25.0 and Microsoft Excel 16, were used to analyze the data. A simple descriptive analysis was carried out, and frequency, means, and percentages were used to present the descriptive results. We checked the data for normality and used the Manne-Whitney U and Kruskal–Wallis tests due to the skewed distribution. The Manne-Whitney U test was used to compare the socio-demographic and obstetric health characteristics between two independent samples, and the Kruskal–Wallis test was used to compare three or more independent samples. In addition, multinomial logistic regression was used for prediction and estimating impact. The Hosmer–Lemeshow goodness of fit test was carried out to check the fitness of the model. All tests were carried out at 95% confidence intervals, and a two-sided significance value (*p*-value) <0.05 was considered statistically significant.

## 3. Results

### 3.1. Socio-Demographic Characteristics

**Figure 1:**
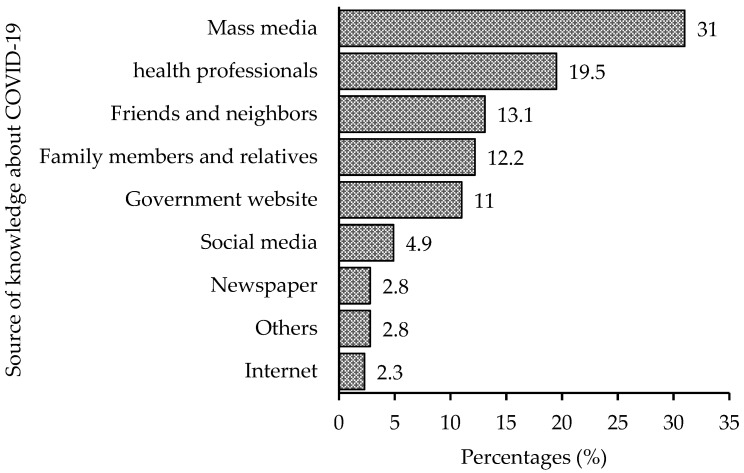
Sources of knowledge regarding COVID-19 among the participants.

The socio-demographic characteristics of our participants are summarized in Table 1. A total of 425 pregnant women participated in this study, with a mean age of 30.37 (SD = 5.12) years. The respondents predominantly resided in rural areas 235 (55.29%) and were Muslim (Islam followers) 357 (84%). Approximately half of the participants, 212 (49.88%) and 160 (37.65%) of the participant’s husbands had minimum higher secondary (11–12) level education. Occupations of the respondents were reported as, 287 (67.53%) homemakers, 78 (18.35%) employees in the private sector, 43 (10.12%) government employees, and 17 (4%) students, or job seekers. Mass media was the primary source of knowledge for the participants (31.06%), followed by health professionals (19.53%), friends and neighbors (13.18%), family members and relatives (12.24%), and government websites (11.06%) (Figure 1).

### 3.2. Obstetric and Reproductive Health Characteristics

The obstetric and reproductive health-related characteristics of our respondents are summarized in Table 1. Our results showed that 276 (64.94%) and 162 (40%) participants were primigravida’s and nulliparas, respectively. Additionally, more than three-fourths of the respondents had no prior history of miscarriage (90.4%, *n* = 384) or abortion (96.9%, *n* = 412), or stillbirth (92.2%, *n* = 392). About 359 (84.47%) respondents reported current pregnancy as wanted and planned. Moreover, 254 (59.76%) participants had been to ANC follow-up for the pregnancy during the study period. Among the participants, 25.18% (*n* = 107) had one child, 6.59% (*n* = 28) had two children or more, and 68.24% (*n* = 290) had no children (Table 1).

### 3.3. Knowledge of Pregnant Women about COVID-19

The results of our survey are summarized in Table 2. According to our study, about half (46.59%, *n* =198) of the participants were aware of the COVID-19 pandemic, that the disease transmits via respiratory droplets of infected individuals (49.18%, *n* = 209) and that this transmission can be prevented by staying indoors, frequent handwashing, and wearing a face mask (51.53%, *n* = 219). The most common knowledge was that they knew the COVID-19 disease was caused by a virus (66.82%, *n* = 284).

More than half of the respondents reported that they did not know the major symptoms of COVID-19 (58.12%, *n* = 247), that the whole population was susceptible to COVID-19 (55.29%, *n* = 235), that pregnant women were at higher risk than others if infected with COVID-19 (54.12%, *n* = 230), and the symptoms appeared after 2–14 days (52%, *n* = 221). They also reported that they did not know that individuals with coronavirus disease can still spread the disease to others without developing signs and symptoms (53.18%, *n* = 226) and they were not aware of vaccine availability (56.94%, *n* = 242). Just 47.76% of the participants had knowledge about COVID-19 that we categorized as “good” (Figure S1).

The results of the comparisons of factors to knowledge are summarized in Table 3. We identified five factors with significant association with knowledge. Place of residence, education level of the participant, occupation of the participant, educational level of the participant’s husband, and ANC visits all showed significant association (*p* < 0.05)) with knowledge score.

Regression analysis revealed three factors associated with the COVID-19 knowledge of the participants. First, participants who lived in a rural area, had lower odds ratio of having knowledge about COVID-19 when compared to urban areas (AOR: 0.59, 95% CI = 0.38–0.92, *p* < 0.05). Second, increasing levels of husband’s education were positively associated with COVID-19 knowledge. Compared to higher education level, when the participant’s husband had secondary and lower levels of education she had a lower odds ratio of knowledge regarding COVID-19 AOR: 0.18, 95% CI = 0.07–0.43, *p* < 0.001), at an intermediate level of education (AOR: 0.32, 95% CI = 0.14–0.71, *p* < 0.01), and a bachelor’s degree (AOR: 0.10, 95% CI = 0.03–0.35, *p* < 0.001). There is a clear positive association between the husband’s level of education and the participant’s knowledge level. Third, participants whose number of ANC visits was less than 3 times (vs. more than or equal to 3 times, AOR: 0.62, 95% CI = 0.39–0.98, *p* < 0.05) had lower odds of knowledge regarding COVID-19 (Table 4).

### 3.4. Attitude of Pregnant Women towards COVID-19

The responses to each question regarding the attitude of pregnant women toward COVID-19 are summarized in Table 2. Just less than half of study respondents agreed that they wanted to reduce or discontinue their prenatal care visits due to COVID-19 (45.41%), and that they feared their new-born might get infected with COVID-19 (46.12%). The response rates of ‘Agree’ were higher for the questions concerning COVID-19 being preventable by practicing social distancing and wearing a facemask (51.06%), and it is treatable at home (48.71%). Overall, 49.41% of the participants had a “positive” attitude toward COVID-19 (Figure S1). Recall that positive is relative to our scoring system and in this sense means that attitude is aligned with scientific evidence, not that there is a warm emotion for the disease.

The results of comparisons of factors to attitude are summarized in Table 3. Four statistically significant associations were found between attitude and sociodemographic variables such as religion, education level of the participant, occupation of the participant, and education level of the participant’s husband (all *p* < 0.05).

The results of the regression analysis of factors to attitude are summarized in Table 4. We identified four significant associations of factors to attitude. First, participants in the age group 25–34 years (vs. ≥35 years, AOR: 0.38, 95% CI = 0.16–0.91, *p* < 0.05) were less likely to have a positive attitude towards COVID-19. Second, the level of education of the participant’s husband affected her attitude, with greater education associated with a more positive attitude. Compared to husbands with higher education, a secondary and lower level of education (AOR: 0.23, 95% CI = 0.09–0.63, *p* < 0.01), and intermediate level of education (AOR: 0.32, 95% CI = 0.13–0.80, *p* < 0.05), were less likely to have a positive attitude towards COVID-19. Third, participants who had only one child (vs. two or more, AOR: 4.87, 95% CI = 1.28–18.51, *p* < 0.05) were more likely to have a positive attitude toward COVID-19. Four, greater knowledge was associated with a more positive attitude. participants having poor knowledge of COVID-19 (vs. good knowledge, AOR: 0.12, 95% CI = 0.07–0.20, *p* < 0.001) were less likely to have a positive attitude toward COVID-19 (Table 4).

### 3.5. Practice of Pregnant Women towards COVID-19

The responses to each practice question are summarized in Table 2. About half of the study participants responded that they did not avoid crowded places during the COVID-19 pandemic (50.12%, *n* = 213), did not maintain a 2 m distance from others (56%, *n* = 238), did not wear a face mask in public (52.47%, *n* = 223), and did not practice frequent handwashing with water and soaps (50.82%, *n* = 216). Overall, 56.24% of the participants had good practice towards COVID-19 (Figure S1). The results of comparisons of factors to practice are summarized in Table 3. We identified four factors with significant association to practice. Religion, education level of the participant, occupation of the participant, and education level of the participant’s husband showed significant associations to practice (all *p* < 0.05), and regression analysis (summarized in Table 4) revealed that participants who had poor knowledge of COVID-19 (vs. good knowledge, AOR: 0.11, 95% CI = 0.07–0.19), *p* < 0.001) were less likely to have good practices towards COVID-19.

## 4. Discussion

This is the first study to investigate the KAP towards COVID-19 among pregnant women in Bangladesh during the COVID-19 pandemic.

Our research, which focused on Northern Bangladesh, revealed that not quite half (46.6%) of the participants had heard about the coronavirus pandemic, comparatively lower than the general Bangladeshi population [23] or pregnant women in Ethiopia [11]. The most credible source of information about COVID-19 for the participants was the mass media (31.1%). This finding is in line with other studies conducted in Ethiopia and Kenya [11,12,24]. This result highlighted the importance of mass media in preventing COVID-19, especially when people are in lockdown. According to our findings, 41.9% of the participants think headache, fever, fatigue, dry cough, and difficulty in breathing were the major clinical symptoms of COVID-19, which is slightly lower compared with recent studies [11,12]. The differences in the results could be explained by the area, context, and survey timing across the countries.

Our study also noted that the participants’ knowledge regarding COVID-19 was higher (47.8%) than in a study in Egypt (16.4%) [25] and similar to studies conducted in Ethiopia [11,12] and South Africa [26]. Nevertheless, it was lower than the findings from other studies conducted in Ghana (85.6%) [9] and India (60%) [10]. The difference, perhaps, is because of the variations in sociodemographic characteristics, study setting, healthcare system, and health education programs of the countries to raise awareness concerning the disease.

Overall, half of the respondents (49.4%) showed a positive attitude towards COVID-19, similar to a study conducted in Ethiopia [12], lower than a study conducted in India [10], and higher than a study carried out in South Africa [26]. The present finding is also lower than studies from Bangladesh [14], Iran [27], and Malaysia [28]. The reasons for these discrepancies might be caused by sociodemographic characteristics, study setting, and study participants. In agreement with the present study, Yassa et al. [29] reported that less than half of the pregnant women do not think that they are more susceptible to getting COVID-19, and their new-born can be infected with COVID-19 compared to non-pregnant women. More importantly, about 45.7% of pregnant women disagreed that social distancing and face masks can play an important role in COVID-19 prevention. The negative attitudes of pregnant women towards COVID-19 could be possible because of the regulatory measures such as lockdowns, social distancing, and use of face masks in public places from the beginning of the confirmed cases in Bangladesh. This negative attitude might mitigate the efforts made by the governments to decrease the spread of COVID-19.

Based on our study findings, 56.2% of the participants had a good level of practice in preventing COVID-19. The finding was higher than a study from Ghana [9] and lower than studies from India [10] and South Africa [26]. However, the study also found that the commonly practiced preventive measures such as wearing a face mask (47.5%), frequent washing of hands with water and soap (49.2%), and maintaining a 2 m distance (44%) were adopted only by less than half of the participants, which was lower than a recent study from Ethiopia [11]. The possible explanation for the low level of adherence to preventive practices could be inadequate face masks, unavailability of soap for handwashing, and high cost or unavailability of hand sanitizer in the study area. In addition, the responses by the participants also exhibited ignorance about the severity of the disease, reluctance to use face masks, and avoid the crowd. This explains the reason behind less compliance by the participants in following precautionary measures specified by the government and maintaining social distancing and other preventive measures.

Similar to the findings of the present study, two other studies on pregnant women revealed that individuals living in urban areas had more knowledge about COVID-19 [9,29]. This is not surprising because urban areas have good infrastructure, such as internet connectivity and other media facilities, compared to their rural counterparts. According to our study, participants’ whose husbands had a higher level of education were found to have more knowledge about COVID-19, whereas other studies found such associations [11,12]. This could be justified by the fact that husbands’ higher level of education leads their wives to access more information technologies with easier access to health information which further helps to educate their wives with basic knowledge of COVID-19. According to the current finding, women who had ANC follow-up during their current pregnancy had a good level of COVID-19 knowledge. The result was supported by a study conducted in Ethiopia [11]. The probable reason might be that participants with ANC follow-up obtained information, main manifestation, and prevention strategies about COVID-19 while visiting healthcare providers for routine obstetric care.

Our study also determines factors associated with pregnant women’s attitudes. Increased age of pregnant women was positively associated with a positive attitude. Recent research conducted in Pakistan concurs with our study results. [30]. The finding might be because the higher the age, the longer the experience dealing with the COVID-19 emergency, showing confidence and optimism. The participants with higher education had a significantly higher positive attitude than participants with lower education. This might be because the educated husband has better access to information via different sources such as newspapers, the Internet, Facebook, and telegram. Moreover, educated individuals also can comprehend the information easily that they receive and follow them. Furthermore, as the educational level of the husband increases, they can learn more accurate information promptly and share more critical topics clearly with their wife. Additionally, an educated husband may better understand complications and outcomes associated with the outbreak, thereby positively influencing his wife’s attitude. Lastly, the pregnant women with good knowledge also showed a good attitude toward COVID-19. This is consistent with a study performed in Ethiopia [12] and Pakistan [30]. The underlying reason might be that good knowledge clears confusion and raises awareness leading to a positive attitude toward COVID-19.

Regarding the practice of pregnant women, it is worth mentioning that higher COVID-19 knowledge scores were found to be associated with a lower likelihood of poor practices towards COVID-19 in this study. Studies from Ethiopia [11] and China [31] are consistent with this study’s findings. This might be because in-depth knowledge about COVID-19 may improve the perception and awareness of the disastrous consequences and, thus, helpful for maintaining good practices to control COVID-19 infection.

This study has several limitations. Due to the scarcity of studies explicitly dedicated to pregnant women, the researchers attempted to refer to other related studies to discuss the results. This may limit the generalizability of the study results. Social desirability and selection bias may also influence the results, as they might deter participants from providing accurate information. Additionally, due to the cross-sectional nature of our study, causal inference cannot be drawn. In the current study, we used only a limited number of questions to assess knowledge, attitude, and practice. Therefore, additional assessments using all aspects of KAP towards COVID-19 would be needed to determine the actual extent of KAP in the general population. However, the findings from our study are useful and are the first to measure the level of KAP of pregnant women towards COVID-19 in Bangladesh.

## 5. Conclusions

In conclusion, our study revealed that about half of the participants had poor knowledge of COVID-19, negative attitudes to COVID-19 science, and poor practices for preventing COVID-19. In addition, women’s age, residence, husband’s educational level, number of living children, and ANC visits were significant predictors of KAP. The most potent predictor of attitudes and practices was knowledge of COVID-19. Thus, a clear avenue has been opened to affect and improve practices in this population to improve knowledge of COVID-19 as well as overall education levels. The authors recommend that the government and other policymakers increase health education and counseling for pregnant women regarding the spread, transmission, and preventive measures to fight against deadly COVID-19. Furthermore, since access to electronic media is limited in rural areas, media campaigns should be extended to rural areas.

## Figures and Tables

**Table 1:** Pregnant women’s distribution by socio-demographic, obstetric, and reproductive health-related characteristics (*n* = 425).

Variables	*N*	Percent (%)
Age, mean, (SD), Year		30.37 (5.12)
**Resident**		
Rural	235	55.29%
Urban	190	44.71%
**Religion**		
Muslim	357	84.00%
Hindu	68	16.00%
**Education**		
No formal education	16	3.76%
Secondary (6–10) and lower	80	18.82%
Intermediate (11–12)	212	49.88%
Bachelor	23	5.41%
Higher Education (Above bachelor)	94	22.12%
**Occupation**		
Housewife	287	67.53%
Employee in the private sector	78	18.35%
Gov. employee	43	10.12%
Student/Job finder	17	4.00%
**Education of Husband**		
Secondary (6–10) and lower	103	24.24%
Intermediate (11–12)	160	37.65%
Bachelor	23	5.41%
Higher Education (Above bachelor)	139	32.71%
**Occupation of Husband**		
Gov. employee	49	11.53%
Employee in the private sector	337	79.29%
Businessman	18	4.24%
Day Labor	21	4.94%
**Gravidity**		
Primi	276	64.94%
Multi	149	35.06%
**Parity**		
Nulliparous	249	58.59%
Primipara	85	20.00%
Multipara	91	21.41%
**Number of Living Children**		
None	290	68.24%
One	107	25.18%
Greater than or equal to 2	28	6.59%
**Condition of Pregnancy**		
Unwanted	54	12.71%
Wanted	359	84.47%
Mistimed	12	2.82%
**ANC Visit**		
No	171	40.24%
Yes	254	59.76%

**Table 2:** Distribution of participants’ KAP towards COVID-19 (*n* = 425).

**Knowledge Items**		**Yes (%)**	**No (%)**
There is an ongoing COVID-19 outbreak.		46.59	53.41
COVID-19 is a disease caused by a virus.		66.82	33.18
COVID-19 spreads via respiratory droplets of infected individuals.		49.18	50.82
COVID-19 can spread through touching the face, mouth, and eyes with the hands of infected individuals.		49.65	50.35
COVID-19 symptoms appear after 2–14 days.		48.00	52.00
The whole population is susceptible to COVID-19.		44.71	55.29
The major clinical symptoms of COVID-19 are headache, fever, fatigue, dry cough, and difficulty in breathing.		41.88	58.12
COVID-19 can affect humans and other animals.		44.00	56.00
COVID-19 is airborne.		44.71	55.29
Staying indoors washing hands frequently and using a face mask can prevent the transmission of COVID-19.		51.53	48.47
COVID-19 symptoms are worse in people with chronic disease.		50.12	49.88
COVID-19 symptoms are worse in older people.		53.41	46.59
Without developing signs and symptoms, individuals with coronavirus disease can spread COVID-19.		46.82	53.18
Pregnant women are at higher risk than others with COVID-19.		45.88	54.12
There is a COVID-19 vaccine available.		43.06%	56.94%
**Attitude Items**	**Disagree (%)**	**Neutral (%)**	**Agree (%)**
COVID-19 is a deadly disease.	44.71	10.59	44.71
People of any age can be infected with COVID-19.	39.53	11.76	48.71
Pregnant women are more susceptible to COVID-19 than non-pregnant women.	40.71	13.88	45.41
COVID-19 makes you reduce or discontinue your routine prenatal care.	42.12	12.00	45.88
Your newborn can be infected with COVID-19.	46.12	7.76	46.12
COVID-19 can be treated at home.	44.71	6.59	48.71
Social distancing and face masks can play an important role in COVID-19 prevention.	45.65	3.29	51.06
**Practice Items**		**Yes (%)**	**No (%)**
Are you obeying government restrictions on COVID-19?		47.29	52.71
Have you stopped going to crowded places during the COVID-19 pandemic?		49.88	50.12
Do you wash your hands frequently using water and soap?		49.18	50.82
Do you maintain 2 m distance from others?		44.00	56.00
Do you avoid touching your eyes, nose, and mouth with unwashed hands?		46.12	53.88
Do you wear a face mask in public?		47.53	52.47
Do you cover your mouth and nose during coughing or sneezing?		55.06	44.94
Do you maintain a healthy lifestyle during COVID-19 outbreak?		43.29	56.71

**Table 3:** Differences in participant’s mean score of COVID-19 related to KAP (*n* = 425).

Categories	*N* (%)	Knowledge		Attitude		Practice	
		(Mean ± SD)	*p* Value	(Mean ± SD)	*p* Value	(Mean ± SD)	*p* Value
**Age**							
15 to 24 Year	55 (12.9)	(7.24 ± 2.2)	0.669	(7.20 ± 2.8)	0.919	(3.67 ± 1.5)	0.565
25 to 34	306 (72.0)	(7.33 ± 2.4)		(7.27 ± 3.1)		(3.82 ± 1.8)	
35 or older	64 (15.1)	(6.98 ± 2.4)		(7.33 ± 3.0)		(3.97 ± 1.6)	
**Resident**							
Rural	235 (55.3)	(6.97 ± 2.3)	0.005	(7.15 ± 2.9)	0.414	(3.84 ± 1.6)	0.774
Urban	190 (44.7)	(7.63 ± 2.4)		(7.42 ± 3.2)		(3.81 ± 1.8)	
Religion							
Muslim	357 (84.0)	(7.20 ± 2.4)	0.156	(7.11 ± 3.0)	0.007	(3.71 ± 1.7)	<0.001
Hindu	68 (16.0)	(7.60 ± 2.4)		(8.13 ± 2.9)		(4.43 ± 1.6)	
**Education**							
No formal education	16 (3.8)	(6.44 ± 2.3)	<0.001	(7.00 ± 3.0)	<0.001	(3.50 ± 1.7)	<0.001
Secondary (6–10) and lower	80 (18.8)	(6.60 ± 2.1)		(6.39 ± 2.7)		(3.31 ± 1.4)	
Intermediate (11–12)	212 (49.9)	(6.93 ± 2.3)		(6.57 ± 2.8)		(3.55 ± 1.6)	
Bachelor	23 (5.4)	(8.78 ± 2.0)		(9.61 ± 2.6)		(4.65 ± 1.7)	
Higher Education (Above bachelor)	94 (22.1)	(8.35 ± 2.5)		(9.09 ± 2.8)		(4.73 ± 1.9)	
**Occupation**							
Housewife	287 (67.5)	(7.10 ± 2.3)	<0.001	(6.96 ± 3.0)	<0.001	(3.63 ± 1.6)	<0.001
Employee in the private sector	78 (18.4)	(7.12 ± 2.5)		(7.33 ± 3.2)		(3.87 ± 1.9)	
Gov. employee	43 (10.1)	(7.91 ± 2.2)		(8.23 ± 2.4)		(4.53 ± 1.6)	
Other	17 (4.0)	(9.06 ± 2.5)		(9.76 ± 2.6)		(5.06 ± 1.7)	
**Education of Husband**							
Secondary (6–10) and lower	103 (24.2)	(6.38 ± 2.0)	<0.001	(6.29 ± 2.5)	<0.001	(3.28 ± 1.4)	<0.001
Intermediate (11–12)	160 (37.6)	(6.88 ± 2.3)		(6.49 ± 2.7)		(3.53 ± 1.5)	
Bachelor	23 (5.4)	(6.22 ± 1.7)		(6.04 ± 3.5)		(3.39 ± 1.8)	
Higher Education (Above bachelor)	139 (32.7)	(8.54 ± 2.4)		(9.10 ± 2.8)		(4.63 ± 1.8)	
**Occupation of Husband**							
Gov. employee	49 (11.5)	(7.67 ± 2.7)	0.355	(8.08 ± 3.5)	0.293	(4.27 ± 1.7)	0.293
Employee in the private sector	337 (79.3)	(7.25 ± 2.4)		(7.18 ± 3.0)		(3.78 ± 1.7)	
Businessman	18 (4.2)	(6.44 ± 1.8)		(6.94 ± 2.5)		(3.67 ± 1.3)	
Student/Job finder	21 (4.9)	(7.29 ± 2.2)		(7.10 ± 2.3)		(3.57 ± 1.5)	
**Gravidity**							
Primi	276 (64.9)	(7.20 ± 2.4)	0.419	(7.21 ± 3.1)	0.603	(3.74 ± 1.7)	0.148
Multi	149 (35.1)	(7.39 ± 2.4)		(7.38 ± 2.8)		(3.97 ± 1.6)	
**Parity**							
Nulliparous	249 (58.6)	(7.18 ± 2.3)	0.948	(7.27 ± 3.2)	0.785	(3.76 ± 1.8)	0.607
Primipara	85 (20.0)	(7.44 ± 2.5)		(7.19 ± 2.8)		(3.96 ± 1.6)	
Multipara	91 (21.4)	(7.33 ± 2.5)		(7.34 ± 2.8)		(3.87 ± 1.6)	
**Number of Living Children**							
None	290 (68.2)	(7.14 ± 2.3)	0.836	(7.15 ± 3.1)	0.223	(3.76 ± 1.7)	0.242
One	107 (25.2)	(7.54 ± 2.6)		(7.67 ± 2.8)		(3.88 ± 1.6)	
Greater than or equal to 2	28 (6.6)	(7.46 ± 2.3)		(7.00 ± 2.8)		(4.25 ± 1.8)	
**Condition of Pregnancy**							
Unwanted	54 (12.7)	(7.44 ± 2.2)	0.731	(7.63 ± 2.9)	0.385	(4.15 ± 1.6)	0.337
Wanted	359 (84.5)	(7.23 ± 2.4)		(7.19 ± 3.1)		(3.78 ± 1.7)	
Mistimed	12 (2.8)	(7.50 ± 1.9)		(8.17 ± 2.7)		(3.67 ± 1.2)	
**ANC Visit**							
No	171 (40.2)	(7.02 ± 2.4)	0.049	(7.14 ± 3.1)	0.376	(3.68 ± 1.7)	0.141
Yes	254 (59.8)	(7.43 ± 2.4)		(7.36 ± 3.0)		(3.92 ± 1.7)	
Total	425 (100.0)	(7.26 ± 2.4)		(7.27 ± 3.0)		(3.82 ± 1.7)	

Note: SD = Standard deviation.

**Table 4:** Multivariable analysis of factors affecting KAP towards COVID-19.

Variables	Knowledge	Attitude	Practice
	AOR (95% CI)	AOR (95% CI)	AOR (95% CI)
**Age**			
15–24	2.34 (0.81–6.76)	0.43 (0.13–1.41)	0.58 (0.19–1.81)
25–34	1.13 (0.52–2.49)	0.38 (0.16–0.91) *	0.68 (0.30–1.57)
35 or older	Ref.	Ref.	Ref.
**Resident**			
Rural	0.59 (0.38–0.92) *	1.15 (0.69–1.90)	1.41 (0.87–2.28)
Urban	Ref.	Ref.	Ref.
Religion			
Muslim	1.07 (0.55–2.07)	1.31 (0.61–2.82)	0.65 (0.32–1.33)
Hindu	Ref.	Ref.	Ref.
**Education**			
No formal education	0.30 (0.07–1.35)	0.84 (0.17–4.29)	0.52 (0.12–2.34)
Secondary (6–10) and lower	0.55 (0.19–1.56)	0.77 (0.24–2.49)	0.68 (0.22–2.09)
Intermediate (11–12)	0.65 (0.26–1.61)	0.56 (0.20–1.58)	0.70 (0.26–1.86)
Bachelor	1.46 (0.40–5.32)	1.43 (0.35–5.93)	1.31 (0.35–4.90)
Higher Education (Above bachelor)	Ref.	Ref.	Ref.
**Occupation**			
Housewife	0.50 (0.11–2.42)	1.02 (0.19–5.40)	0.59 (0.10–3.34)
Employee in the private sector	0.28 (0.06–1.36)	0.81 (0.15–4.31)	0.56 (0.10–3.22)
Gov. employee	0.72 (0.14–3.82)	1.22 (0.21–7.17)	0.98 (0.15–6.28)
Student/Job finder	Ref.	Ref.	Ref.
**Education of Husband**			
Secondary (6–10) and lower	0.18 (0.07–0.43) ***	0.23 (0.09–0.63) **	1.08 (0.41–2.87)
Intermediate (11–12)	0.32 (0.14–0.71) **	0.32 (0.13–0.80) *	1.30 (0.52–3.24)
Bachelor	0.10 (0.03–0.35) ***	0.38 (0.10–1.41)	2.07 (0.58–7.40)
Higher Education (Above bachelor)	Ref.	Ref.	Ref.
**Occupation of Husband**			
Gov. employee	0.85 (0.24–3.00)	1.28 (0.31–5.27)	3.16 (0.84–11.91)
Employee in the private sector	1.05 (0.35–3.20)	1.19 (0.34–4.16)	1.58 (0.50–4.97)
Businessman	0.73 (0.16–3.30)	1.67 (0.34–8.15)	3.94 (0.81–19.14)
Daily laborer	Ref.	Ref.	Ref.
**Gravidity**			
Primi	1.33 (0.47–3.72)	1.66 (0.54–5.10)	0.89 (0.32–2.46)
Multi	Ref.	Ref.	Ref.
**Parity**			
Nulliparous	1.55 (0.53–4.55)	1.50 (0.46–4.83)	1.02 (0.33–3.12)
Primipara	1.83 (0.80–4.16)	1.01 (0.41–2.51)	1.66 (0.70–3.98)
Multipara	Ref.	Ref.	Ref.
**Number of Living Children**			
None	0.48 (0.10–2.23)	1.83 (0.33–10.11)	0.51 (0.11–2.40)
One	1.10 (0.35–3.40)	4.87 (1.28–18.51) *	0.57 (0.17–1.89)
Greater than or equal to 2	Ref.	Ref.	Ref.
**Condition of Pregnancy**			
Unwanted	0.59 (0.13–2.55)	0.64 (0.12–3.39)	1.90 (0.42–8.57)
Wanted	0.72 (0.18–2.95)	0.53 (0.11–2.60)	2.63 (0.64–10.77)
Mistimed	Ref.	Ref.	Ref.
**ANC Visit**			
No	0.62 (0.39–0.98) *	1.28 (0.77–2.14)	0.98 (0.60–1.58)
Yes	Ref.	Ref.	Ref.
**Knowledge**			
Poor knowledge	-	0.12 (0.07–0.20) ***	0.11 (0.07–0.19) ***
Good knowledge	-	Ref.	Ref.

Note: * *p* < 0.05; ** *p* < 0.01; *** *p* < 0.001, AOR = Adjusted Odds Ratio, CI = Confidence Interval.

## Data Availability

The dataset used and analyzed during the current study is available from the corresponding author on request.

## Supplementary Materials

The following supporting information can be downloaded at: https://www.mdpi.com/article/10.3390/bs13010002/s1, Figure S1: Level of KAP regarding COVID-19 among the participants.
