# Supplementary figures and images for: Knowledge, Attitudes, and Practices towards COVID-19 among Pregnant Women in Northern Bangladesh: A Community-Based Cross-Sectional Study
Source: Behav Sci (Basel). 2022 Dec 20;13(1):2. doi: 10.3390/bs13010002 (PMC9855099; doi:10.3390/bs13010002)

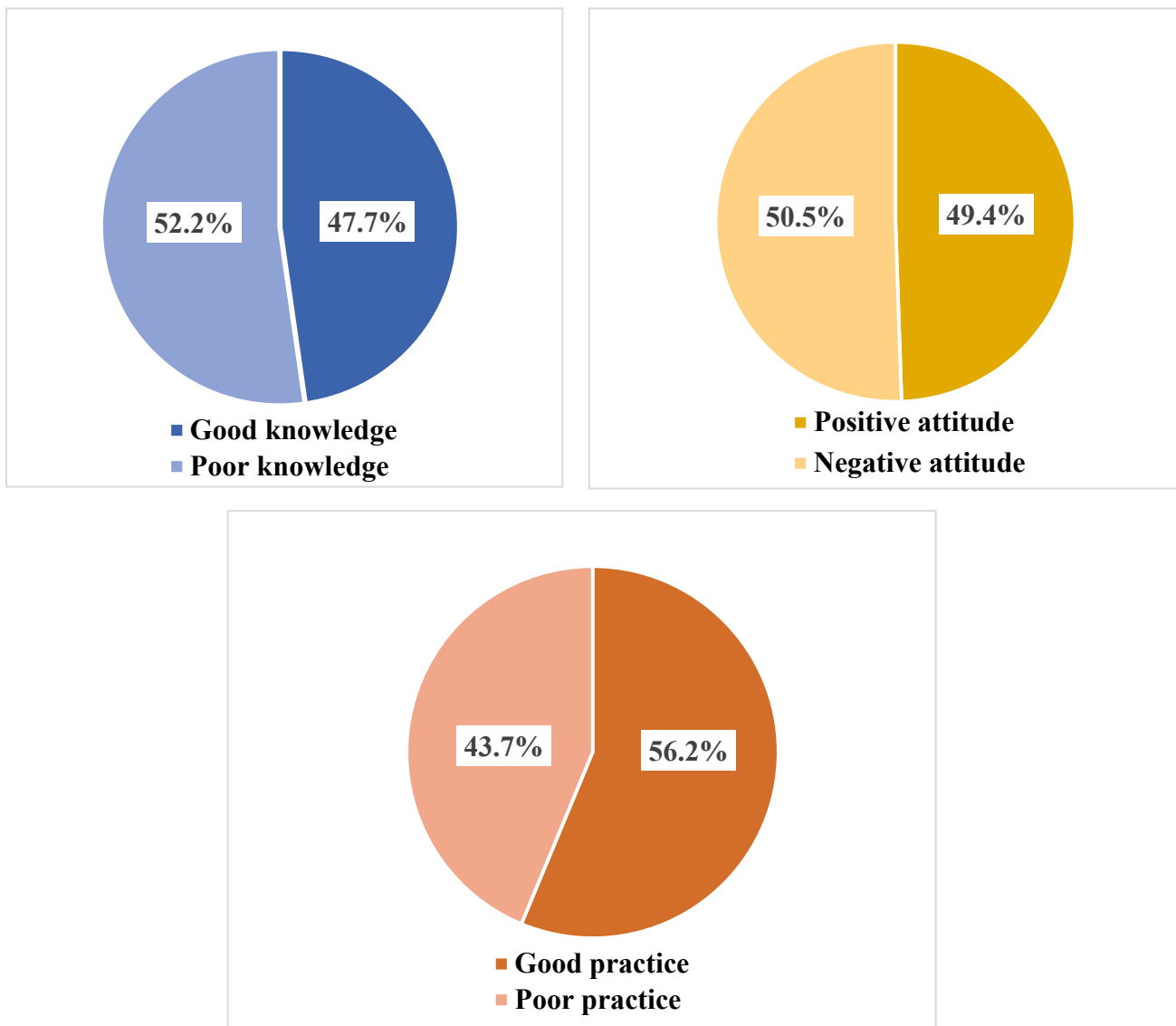

**Figure S1.** Level of KAP regarding COVID-19 among the participants

Supplement: Supplementary file 1 [file behavsci-13-00002-s001.zip › behavsci-2089755-supplementary.pdf]
